# Characterization of the complete chloroplast genome of *Sanicula rubriflora F. Schmidt ex Maxim*

**DOI:** 10.1080/23802359.2021.1938728

**Published:** 2021-06-14

**Authors:** Zhen Wang, Weichao Ren, Song Yan, Meiqi Zhang, Yunwei Liu, Wei Ma

**Affiliations:** a Pharmacy College, Heilongjiang University of Chinese Medicine, Harbin, China; b State key laboratory of tree genetics and breeding, Northeast Forestry University, Harbin, China; c Yichun Branch of Heilongjiang Academy of Forestry, Yichun, China

**Keywords:** *Sanicula rubriflora*, complete chloroplast genome, phylogeny, Apiaceae

## Abstract

*Sanicula rubriflora* refers to a kind of edible wild herbs, which possesses reputed medicinal value. Chloroplasts (cp) is essential organelle in plant cells and has a genome that can be inherited autonomously. The complete chloroplast genome of *S. rubriflora* was assemble and annotated in the present study. It was a circular molecular genome with a size of 155,700 bp in length, which has a typical quadripartite structure. For instance, a large single-copy (LSC) of 85,979 bp and a pair of inverted repeats (IRs) of 26,333 bp were disconnected by a small single-copy (SSC) of 17,053bp. A total of 130 genes have been annotated, including 86 protein-coding genes (PCGs), 36 transfer RNA genes (tRNAs) and 8 ribosomal RNA genes (rRNAs). The total GC content of the complete chloroplast genome reached 37.9%. A maximum likelihood phylogenetic analysis with the reported chloroplast genomes revealed that *S. rubriflora* is most closely related to *Saniculachinensis* in the phylogenetic relationships.

*Sanicula rubriflora F. Schmidt ex Maxim.* is a perennial herb in the Apiaceae family. It is an edible mountain potherb with approximately 40 species worldwide (Wyk et al. 2013). It grows in the mountain forests with moist humus, having altitude of 200–470 m. The main production areas are in Northeast China, and distributed in Mongolia, North Korea and northern Japan (Vandelook and Van Assche 2008). Chloroplasts are commonly found in terrestrial plants, algae and some protists. They are photosynthetic organelles of green plants, and their functions include the production of pigments, synthesis of sugars and some amino acids (Xing 2008). In angiosperms, the chloroplast genome is relatively conserved, and most of which are double-stranded loop structures, including a small single copy (SSC), a large single copy (LSC), and two sequences with the same code and opposite directions inverted repeats sequence (IR) (Zhang et al. 2012). In comparison with the nuclear genome, the chloroplast genome has multiple advantages such as relatively conservative structure, moderate base mutation rate, and easy sequencing with wide application in the study of systematic evolution of various plant groups (Daniell et al. 2016).

Fresh leaves of *S. rubriflora* was collected from Yichun City, Heilongjiang Province, China on 20 August 2020 (N:47°81'08″, E:128°90'97″). The samples are kept in Heilongjiang University of Traditional Chinese Medicine (YCL20190507007). The total genomic DNA of *S. rubriflora* was isolated by CTAB methods, and genomic DNA was stored at Heilongjiang University of Traditional Chinese Medicine. The whole genome sequencing was carried out on Illumina Hiseq platform by Benagen Tech Solutions Co., Ltd (Wuhan, China) with SOAPnuke software for low-quality reads filtering and delete low-quality reads and adapters for deletion. The complete chloroplast genome sequence of *S. rubriflora* was assembled and annotated with the application of SPAdes software (Bankevich et al. 2012). The complete chloroplast genome was submitted to GenBank with the accession number of MW690208.

*Sanicula rubriflora* complete chloroplast genome was a quadripartite structure with 155,700 bp in length, which was composed of four distinct regions including a large single-copy (LSC) region of 85,979 bp, a small single-copy (SSC) region of 17,053 bp and two inverse repeat sequences IRa and IRb (26,333 bp). The overall nucleotide composition is A of 30.61%, T of 31.22%, C of 19.4% and G of 18.78%. In addition, a total of 130 genes are encoded containing 86 protein-coding genes (PCGs), 36 transfer RNA genes (tRNAs) and 8 ribosomal RNA genes (rRNAs).

In order to research the position of *S. rubriflora* in phylogeny, we collected 13 chloroplast genomes and two taxa (*Arabidopsis thaliana* and *Zea mays*) as outgroups with sequenced chloroplast genomes. Sequences were aligned with MAFFT (John et al. 2019). The maximum likelihood (ML) tree was performed demonstrated MEGA X (Sudhir et al. 2018) software by 1000 bootstrap replicates (Figure 1). The ML tree analysis demonstrated that *S. rubriflora* was closely related to *Sanicula chinensis*. The chloroplast genome of *S. rubriflora* provides more detailed and complete data for the study of *Sanicula*, thus laying the foundation for the identification of the genus and the analysis of genetic differences at the population and individual levels.

**Figure 1.**
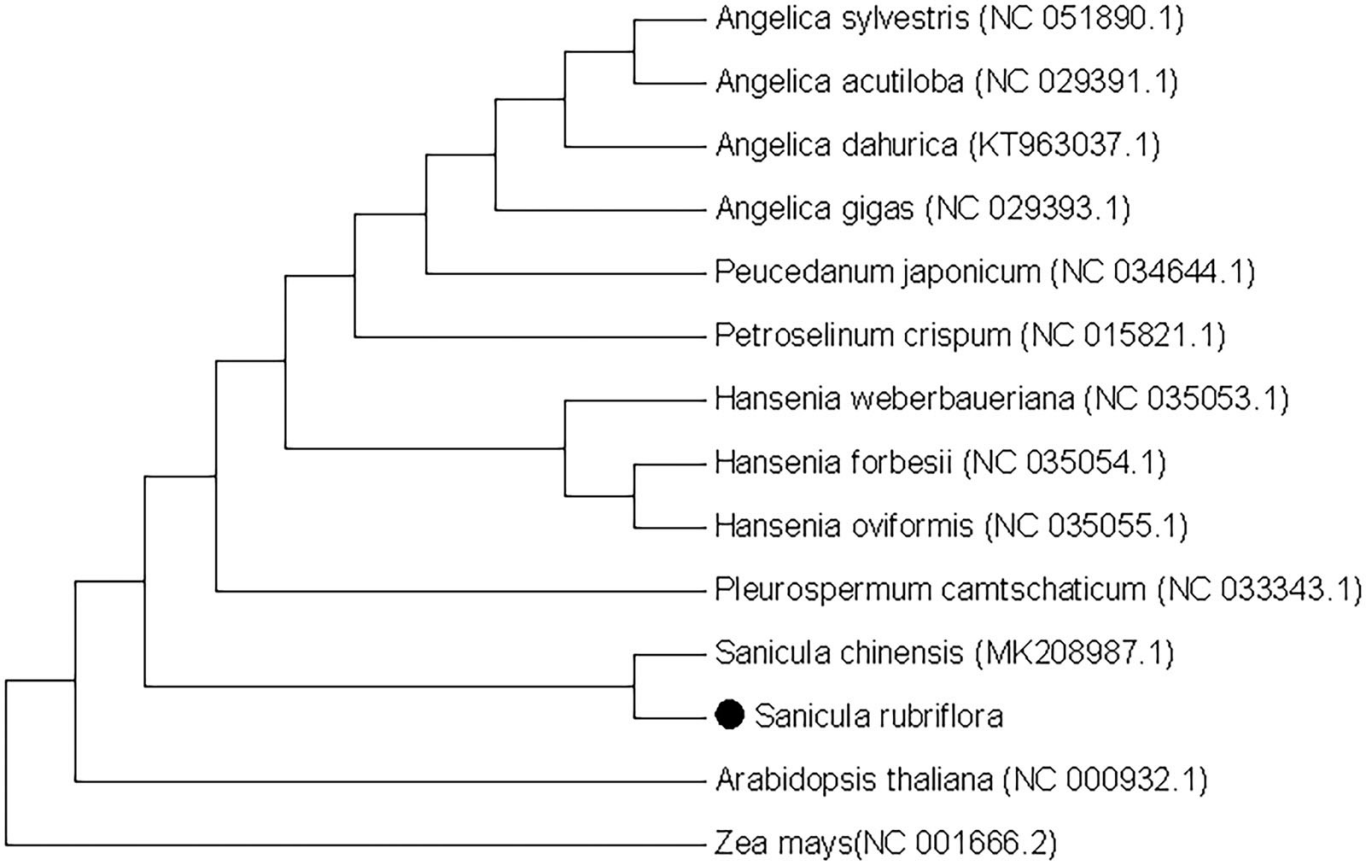
Phylogenetic tree reconstruction of 14 samples using maximum likelihood based on complete chloroplast genome. Note: Sequence data was obtained from NCBI; •represents the species in this study.

## Data Availability

The genome sequence data that support the findings of this study are openly available in GenBank of NCBI at (https://www.ncbi.nlm.nih.gov/) under the accession no. MW690208.

## References

[CIT0001] Bankevich A, Nurk S, Antipov D, Gurevich AA, Dvorkin M, Kulikov AS, Lesin VM, Nikolenko SI, Pham S, Prjibelski AD, et al. 2012. SPAdes: a new genome assembly algorithm and its applications to single-cell sequencing. J Comput Biol. 19(5):455–477. 22506599 10.1089/cmb.2012.0021 PMC3342519

[CIT0002] Daniell H, Lin CS, Yu M, Chang WJ. 2016. Chloroplast genomes: diversity, evolution, and applications in genetic engineering. Genome Biol. 17(1):1–29. 27339192 10.1186/s13059-016-1004-2 PMC4918201

[CIT0003] John R, Songling L, Mar AK, Standley DM, Kazutaka K. 2019. MAFFT-DASH: integrated protein sequence and structural alignment. Nucleic Acids Res. 47(W1):W5–W10. 31062021 10.1093/nar/gkz342 PMC6602451

[CIT0004] Sudhir K, Glen S, Michael L, Christina K, Koichiro T. 2018. MEGA X: molecular evolutionary genetics analysis across computing platforms. Mol Biol Evol. 35(6):1547–1549. 29722887 10.1093/molbev/msy096 PMC5967553

[CIT0005] Vandelook F, Van Assche JA. 2008. Deep complex morphophysiological dormancy in *Sanicula europaea* (Apiaceae) fits a recurring pattern of dormancy types in genera with an Arcto-Tertiary distribution. Botany. 86 (12):1370–1377.

[CIT0006] Wyk BEV, Tilney PM, Magee AR. 2013. African Apiaceae: a synopsis of the Apiaceae/Umbelliferae of sub-Saharan Africa and Madagascar. Pretoria: Briza Academic Books.

[CIT0007] Xing SC. 2008. Process in chloroplast genome analysis. Progress Biochem Biophys. 35(001):21–28.

[CIT0008] Zhang T, Fang Y, Wang X, Deng X, Zhang X, Hu S, Yu J. 2012. The complete chloroplast and mitochondrial genome sequences of *Boea hygrometrica*: insights into the evolution of plant organellar genomes. PLOS One. 7(1):e30531. 22291979 10.1371/journal.pone.0030531 PMC3264610

